# Diagnosis and treatment of pancreatic metastases in 22 patients: a retrospective study

**DOI:** 10.1186/1477-7819-12-299

**Published:** 2014-09-25

**Authors:** Shao-Wei Song, Jun-Feng Cheng, Ning Liu, Ting-Han Zhao

**Affiliations:** Department of General Surgery, the First Hospital of China Medical University, 155 North Nanjing Street, Heping District, Shenyang, 110001 China

**Keywords:** Pancreatic metastasis, Diagnosis, Treatment, Initial symptom, Retrospective analysis

## Abstract

**Background:**

Pancreatic metastases (PMs) are rare and lack of guidelines for diagnosis and treatments .The aim of this study is to explore the diagnosis, treatment, and prognosis of pancreatic metastases.

**Methods:**

Twenty-two patients with pancreatic metastases who had been hospitalized at the First Affiliated Hospital of China Medical University from October 1980 to October 2012 were included in the present retrospective study. Seven patients had gastric cancer, five had colon cancer, two each had lung and liver cancer, and one each had bladder cancer, gallbladder cancer, breast cancer, nasopharyngeal cancer, renal cell carcinoma, and carcinoid.

**Results:**

No specific syndrome or imageological change was found for the pancreatic metastases. The most common symptoms were abdominal pain and jaundice. Hypo-echoic lesions with well-defined margins were found on ultrasonic examinations, and low-density lesions with heterogeneous enhancement were identified in CT images. Nineteen of the 22 received treatment. Three of the 8 patients (34.1%) that had undergone operation experienced complications, but all patients recovered after conventional treatment. Follow-up studies were performed for 17 patients (77.3%), and the median survival time from the diagnosis of pancreatic metastases was 13.2 months (range, 2 to 68 months). Of the five patients who underwent radical resection, one was lost to follow-up, one died at fifteen months postoperation, and the other three are still alive and free from disease (disease-free survival ranging from five to thirty-three months from the diagnosis of the pancreatic metastases).

**Conclusion:**

Pancreatic metastases are rare lesions with no specific symptoms. Radical resection should be performed if possible; however, aggressive treatment should be performed for unresectable pancreatic metastases.

## Background

Most pancreatic cancers are primary tumors, and pancreatic metastases (PMs) are actually quite rare, with only 2% of pancreatic cancers representing PMs [1]. Moreover, most PMs are accompanied by additional metastases outside of the pancreas. No guidelines have been developed for the diagnosis and treatments of PMs; therefore, the appropriate treatment for PMs remains controversial. Here, we retrospectively analyzed the clinical data from 22 patients with PMs to investigate their diagnosis and treatment.

## Methods

### General information

The characteristics of the 22 patients included in this study are shown in Table 1. Among these patients, there were 13 men and 9 women. The median age of the patients was 61 years (range, 43 to 86 years). Lesions at the head and neck of the pancreas were found in 17 patients, while lesions at the tail of the pancreas were found in 5 patients. Twenty patients had a single tumor, while two patients had multiple tumors. No diffuse enlargement of the pancreas was found. The mean longest diameter of the tumors was 3.07 cm (range, 1.2 to 10.0 cm).

**Table 1:** **Characteristics of the 22 patients**

Parameter	Value
Number	22
Male, n (%)	13 (59.1%)
Median age (interquartile range)	61 (52 to 65) years
Primary tumor, n (%)	
Gastric cancer	7 (31.8%)
Colon cancer	5 (22.7%)
Liver cancer	2 (9.1%)
Lung cancer	2 (9.1%)
Others	6 (27.3%)
Longest diameter of the tumor (mean)	1.2 to 10.0 (3.07) cm
Location of the tumor	
Pancreatic head	17
Pancreatic body or tail	5
Single tumor, n (%)	20 (90.9%)
Time between the diagnosis of primary tumor and PMs (mean)	1 to 192 (55) months
Patients with symptoms, n (%)	18 (81.8%)
Initial symptom abdominal pain	9 (50%)
Jaundice	8 (44.4%)
Others	1 (5.6%)

PMs, pancreatic metastases.

### The primary tumor and time from diagnosis of primary tumor to PMs

The primary tumors of the twenty-two patients with PMs were as follows: gastric cancer in seven patients, colon cancer in five patients, lung cancer in two patients, liver cancer in two patients, bladder cancer in one patient, gallbladder cancer in one patient, nasopharyngeal cancer in one patient, renal cell carcinoma in one patient, breast cancer in one patient, and carcinoid in one patient. The mean time between the diagnosis of the primary tumor and PMs was 55 months (range, 1 to 192 months). The longest time between the diagnosis of primary tumor and PMs was found in a patient that had been diagnosed with colon cancer sixteen years before the diagnosis of PMs, while the primary tumor and PMs were diagnosed at the same time in the other five patients (two with primary colon cancer, one with gastric cancer, one with carcinoid, and one with lung cancer).

### Diagnostic methods for PMs

For the twenty-two patients with PMs, five patients were diagnosed by pathological examinations, five were diagnosed by cytological examinations using fine-needle aspirate biopsy, and the other patients were diagnosed by clinical symptoms, imaging examinations (including ultrasonic examination, computed tomography (CT), magnetic resonance imaging (MRI), and positron emission tomography-CT (PET-CT), and history of malignancies.

## Results

### Pancreatic metastasis-related initial symptoms

No specific symptoms were found for the patients with PMs. The major initial symptoms of these patients were jaundice (9 patients, 50%) and discomfort or pain in the upper abdomen (8 patients, 44.4%). The initial symptom of one case was acute pancreatitis, and the other symptoms included gastrointestinal hemorrhage, nausea, and vomiting. For four other patients, PMs were diagnosed when patients came to the hospital for re-examination of the primary tumors but were asymptomatic.

### Imaging findings

Ultrasonic examination was performed for 19 patients, and the most common findings were heterogeneous or homogeneous hypo-echoic lesions with well-defined margins and mild expansion of the pancreatic ducts. CT scanning was performed for 18 patients and revealed low-density lesions in the pancreas for most of the patients. The metastases of sixteen of the 18 patients who received CT scanning were also found without enhancement or with mild or heterogeneous enhancement, but the CT numbers were still lower than the pancreatic parenchymal density even after the enhancement. Another patient with clear cell renal cell carcinoma as the primary tumor was found to have substantial enhancement. MRI examination found no obvious abnormalities in one patient; however, another patient was found with an equisignal on T1WI and mildly increased signal on T2WI. PET-CT scanning showed significantly increased ^18^flourodeoxyglucose (FDG) uptake in the lesions within the pancreas.

### Treatments

Nineteen of the 22 patients received treatment, while the other 3 patients refused treatment. Eight of the nineteen patients who requested treatment underwent surgery, including radical resection of the tumor for five patients (four received pancreaticoduodenectomy and one with primary colon cancer received right hemicolectomy and resections of the spleen, pancreatic body, and pancreatic tail) and palliative operation for three patients. Four of the nineteen patients received interventional operation, including biliary stent implantation for three patients, pancreatic arterial infusion chemotherapy for one patient, and radiotherapy or chemotherapy for seven patients.

### Survival

We called all patients to follow-up in December 2009 and obtained information from 17 patients (77.27%). The median survival time of these 17 patients was 13.2 months (range, 2 to 68 months). For the five patients who received radical resection, the disease-free survival was five to thirty-three months for three patients after treatment. The longest survival time (68 months) was found in a patient with primary colon cancer.

## Discussion

Pancreatic metastases (PMs) are very rare, accounting for only about 2% of malignancies of the pancreas [1]. In a large autopsy series, the prevalence of PMs was reported to be as high as 6 to 11% [2]. Studies have reported that people of about 60 years old are at high risk of developing PMs; however, no significant gender difference has been reported [3], which is in agreement with the findings of the present study (median age, 61.5 years; male/female ratio of 1.4:1). Renal cell carcinoma appears to be the most common primary tumor to cause secondary pancreatic tumors for people outside of China [3, 4]. In Chinese individuals with PMs, lung cancer (especially small cell lung cancer) has been reported to be the most common primary tumor [5–7], followed by gastric and colon cancers. Renal cell carcinoma accounts for very few patients with PMs in China. We believe that the widespread smoking in China could cause increased risk of lung cancer, and small cell lung cancer can develop very quickly, thus permitting the rapid formation of distal metastases. The findings of the present study showed that most patients with PMs had primary gastric cancer or colon cancer, which could partly be affected by the regional distribution of different cancers.

Generally, primary cancers require a long time to spread to the pancreas, especially for renal cell carcinoma. Studies have reported that the median time for renal cell carcinoma to spread to the pancreas is 13 years, while that for other cancers is 4 years [8]. For some special cases, PMs could develop with the primary tumors almost simultaneously [9]. The mean time between the diagnosis of primary tumor and PMs was 55 months in the present study, which is consistent with previous reports.

No specific symptoms or imaging features were found for PMs. Studies [4] have reported that the most common symptoms of PMs include abdominal pain, jaundice, and gastrointestinal hemorrhage. In addition, for patients with primary lung cancer, acute pancreatitis could be the initial symptom. The main feature on ultrasonic examination is hypo- or mixed echoic lesions [5]. The main feature in CT imaging is low-density lesions without enhancement or with heterogeneous enhancements; however, for patients with substantial enhancements in the lesion and a history of renal carcinoma, renal cell carcinoma should be strongly considered as the primary tumor of the PMs. In the present study, pathological examination showed that one patient had PMs that had spread from renal cell carcinoma, and the CT image revealed a large blood supply, while a smaller blood supply was generally found in other patients. Other features, including double duct signs, peripancreatic infiltration, and distant metastasis, could also be found. MRI examinations generally reveal hypo- or equisignals on T1WI images and hypersignals or mildly increased signals on T2WI images; however, the signals are generally heterogeneous. Enhanced scanning generally reveals ring-enhancement, and the gaps between the peripancreatic fat tissues could be displayed clearly with mild expansion of the pancreatic ducts, but without invasion of adjacent vessels [10]. Only very few studies have reported the features of PET-CT images of PMs. However, PET-CT could effectively display micrometastases all over the body, which could help in choosing the appropriate treatment method. In the present study, two patients were examined with PET-CT and were found to have lesions exhibiting increased FDG uptake, but no other abnormalities. These two patients were successfully treated with radical surgery. Postoperative pathological examination is essential for the diagnosis of PMs. In the present study, five patients were diagnosed by postoperative pathological examinations, another five patients were diagnosed by cytological examinations using fine-needle aspirate biopsy; the other patients were diagnosed by clinical symptoms, imaging presentations, and history of malignancies. Previous disease history plays a critical role in the differential diagnosis, while cytological examination by fine-needle aspirate biopsy is not recommended as a routine pre-operative examination as it could possibly introduce peritoneal dissemination of the tumor [11].

The treatment for PMs remains controversial. Since most patients with PMs are at advanced stages and generally with systemic metastasis, operation is not appropriate. Fortunately, the advances made in pancreatic surgery in recent years have greatly increased the safety of the operation; this has extended the indications for surgery, and patients with PMs can now also be treated this way. However, no guidelines on the treatment of patients with PMs have been issued to date, except for a single paper, focused on the surgical treatment of PMs, which was published in *The Lancet*[4] in 2009 and which provided valuable information on treating PMs.

According to the guidelines issued by Reddy and Wolfgang and other studies [3], several characteristics are required for treating PMs with radical surgery: first, the primary cancer type should be associated with successful outcomes; second, the primary cancer site has been well controlled; third, the PMs should be proven as isolated metastases; fourth, the PMs should not involve invasion of the adjacent vessels, and the clinicians should consider the resectability of the metastasis; and fifth, the patient should be expected to be able to tolerate the operation. However, although invasion of the peripancreatic veins is not a contraindication for the operation, clinicians should be very careful in treating such patients. In the present study, all patients who received radical surgery had isolated metastases, and the primary cancer sites in these patients had been well controlled. Follow-up studies revealed no signs of recurrence, and the patients generally had excellent long-term outcomes.

Currently, researchers believe that radical surgery should be performed in treating isolated single PMs as often as possible [1, 8, 9, 11]; however, the treatment of isolated multiple PMs still remains controversial. Several researchers have proposed that total pancreatectomy should be performed for these patients because multiple PMs could involve the entire pancreas. In contrast, other researchers believe that such patients should not be treated surgically because the presence of multiple PMs indicates fatal generalized dissemination [12]. Interestingly, Sellner *et al*. [13] have reported that patients with single PMs or isolated multiple PMs have similar three- and five-year survival rates; thus, radical operation could be performed for patients with isolated multiple PMs. In addition, studies have demonstrated that patients are at a high risk of developing diabetes after total pancreatectomy. Therefore, Bassi *et al*. [14] performed irregular or subtotal pancreatic resection when treating such patients; however, a high incidence of complications and high local recurrence rates were reported.

For some cases in which extensive tumor metastases have been found during the operation and in which pancreatic resection could not be performed, palliative surgery, including Roux-en-Y biliary-jejunal anastomosis, gastrojejunostomy, and biliary stent implantation, should be the treatment of choice to remove the obstruction from the biliary tract and duodenum, which could also improve patient quality of life. In the present study, two patients received biliary-intestinal anastomosis, one received cholecystostomy, and four received biliary stent implantation for the treatment of obstructive jaundice.

Comprehensive treatment, including radiotherapy and chemotherapy, should be the treatment of choice for patients with PMs and systemic metastasis. However, different treatment methods should be performed for different patients depending on the primary tumor. In brief, chemotherapy should be performed for PMs patients with primary lung cancer, especially small cell lung cancer, which could result in early metastasis. In patients with renal cell carcinoma that has spread to the pancreas, treatment with IFN-α and IL-2 used to be common; however, several new drugs, including temsirolimus, bevacizumab, sunitinib, and sorafenib, have proven to be effective in treating such diseases. Moreover, the latter two drugs, namely sunitinib and sorafenib, have been approved by the Food and Drug Administration (FDA) as second-line treatments that should be used if the patients do not respond to cytokine therapy. These new drugs are also applicable for patients with advanced renal cell carcinomas that have spread to the pancreas, but who could not be treated surgically. In recent years, researchers have highlighted the importance of systemic comprehensive treatments in treating pancreatic metastases from renal cell carcinoma, and operative treatment should only be considered as a part of multimodal therapy or combination treatment instead being the sole treatment method [15, 16]. We believe that further studies should be performed to investigate the most appropriate comprehensive treatment for unresectable PMs.

**Figure 1:**
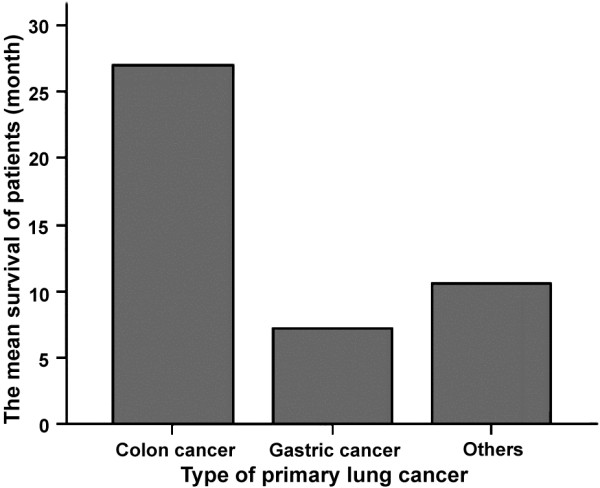
**Comparison of colon cancer and gastric cancer prognosis.**

Generally, the outcomes of PMs are better than those of primary pancreatic cancers [11], and several other factors affect these outcomes. Many studies [17–19] have demonstrated that the most important factor affecting the outcome of PMs is the pathological type of the primary tumor. The best and worst outcomes have been found in patients with primary renal cell carcinoma and lung cancer, respectively. The 5-year survival rate for patients with primary renal cell carcinoma is 29%, and the median survival time is 54 months; for the patients with primary lung cancer, the median survival time is only 6 months [4, 5, 19]. In the present study, the median survival time was 27 months from the diagnosis of PMs for patients with primary colon cancer, and the outcomes of these patients were better than those in patients with gastric cancer (Figure 1). The mean disease-free survival of patients with primary renal cell carcinoma was 16 months from the operation. The longest survival time was found in a patient with primary colon cancer that had spread to the pancreas after operation. For this patient, pancreatic artery perfusion therapy has been performed regularly, and the patient has survived for more than 68 months with the tumor; examinations have also revealed signs of tumor regression. Several studies have suggested that radical surgery is a favorable factor for the prognosis of patients with PMs [13, 18]. In the present study, better long-term outcomes were found in patients who received radical surgery. In brief, among the five patients who were treated with radical surgery, one died at fifteen months postoperation, one was lost to follow-up, and the other three patients are living disease-free at the time of writing this manuscript, giving a mean survival time of 18 months after the surgical treatment of PMs. In contrast, the mean survival time of patients who were treated with palliative treatment and follow-up was only 6.8 months after the diagnosis of PMs. In Table 2,we showed the recent published series of patients with pancreatic metastases.

**Table 2:** **Recent published series of patients with pancreatic metastases**

Author, year	Number of patients	Metastasis from lung cancer	Metastasis from renal cancer	Metastasis from gastric cancer	Incidence	Interval (month)	Symptoms at diagnosis	Synchronous metastasis	Survival (months)	
									2 year	5 year
Present study	22	2 (9.1% )	1 (4.5%)	7 (31.8%)	2%	55 (1 to 92)	18 (81.8%)	2 (9.1% )	35.3%	11.8%
Markinez [20], 2013	8	0	8 (100%)	0	1.2%	149 (19 to 361)	NR	3 (37.5%)	37.5%	12.5%
Boo SJ [21], 2011	31	9 (29%)	16 (51.6%)	1 (3.2%)	NR	40.8 (3 to 186)	NR	6 (19.4%)	NR	NR
Reddy S [17], 2008	49	4 (8.2%)	21 (42.9%)	0	NR	4.8	45 (92%)	7 (14%)	NR	NR
Zerbi A [22], 2008	36	0	36 (100%)	0	NR	8	8 (22%)	0	95%	88%
Sellner F [13], 2006	236	0	236 (100%)	0	NR	10	66 (65%)	23 (12%)	78%	72%
Xu DK [5], 2006	18	8 (44.4%)	1 (5.6%)	2 (11.1%)	15%	12 (3 to 216)	NR	4 (22.2%)	NR	NR

NR: not reported.

## Conclusions

Pancreatic metastases are rare lesions without specific symptoms. Radical resection should be performed if possible; however, multiple treatment should be performed for unresectable pancreatic metastases. However, the sample size of the present study was small, and further studies are needed to validate our findings and to identify the factors that affect the prognosis of PMs.

## Abbreviations

PMs: pancreatic metastases
CT: computed tomography
MRI: magnetic resonance imaging
PET-CT: positron emission tomography-CT
FDG: flourodeoxyglucose
FDA: Food and Drug Administration.
